# Eurasian Tree Sparrows, Risk for H5N1 Virus Spread and Human Contamination through Buddhist Ritual: An Experimental Approach

**DOI:** 10.1371/journal.pone.0028609

**Published:** 2011-12-02

**Authors:** Ramona Alikiiteaga Gutiérrez, San Sorn, John M. Nicholls, Philippe Buchy

**Affiliations:** 1 Virology Unit, Institut Pasteur in Cambodia, Phnom Penh, Cambodia; 2 National Veterinary Institute, Ministry of Agriculture Forestry and Fisheries, Phnom Penh, Cambodia; 3 Department of Pathology, University of Hong Kong, Pok Fu Lam, Hong Kong, People's Republic of China; Centers for Disease Control and Prevention, United States of America

## Abstract

**Background:**

The Highly Pathogenic Avian Influenza H5N1 virus has dramatically spread throughout Southeast Asia since its first detection in 1997. Merit Release Birds, such as the Eurasian Tree Sparrow, are believed to increase one's positive karma when kissed and released during Buddhist rituals. Since these birds are often in close contact with both poultry and humans, we investigated their potential role in the spread of H5N1 virus.

**Methodology/Principal Findings:**

Seven series of experiments were conducted in order to investigate the possible interactions between inoculated and exposed birds, including sparrow/sparrow, sparrow/chicken, duck/sparrow. Daily and post-mortem samples collected were tested for H5N1 virus by real-time RT-PCR and egg inoculation. When directly inoculated, Eurasian Tree Sparrows were highly susceptible to the H5N1 virus, with a fatality rate approaching 100% within 5 days post-inoculation. Although transmission of fatal infection between sparrows did not occur, seroconversion of the exposed birds was observed. Up to 100% chickens exposed to inoculated sparrows died of H5N1 infection, depending on the caging conditions of the birds, while a fatality rate of 50% was observed on sparrows exposed to infected ducks. Large quantities of H5N1 virus were detected in the sparrows, particularly in their feathers, from which infectious particles were recovered.

**Conclusions/Significance:**

Our study indicates that under experimental conditions, Eurasian Tree Sparrows are susceptible to H5N1 infection, either by direct inoculation or by contact with infected poultry. Their ability to transmit H5N1 infection to other birds is also demonstrated, suggesting that the sparrows may play a role in the dissemination of the virus. Finally, the presence of significant quantities of H5N1 virus on sparrows' feathers, including infectious particles, would suggest that Merit Release Birds represent a risk for human contamination in countries where avian influenza virus is circulating and where this religious ritual is practiced.

## Introduction

The Highly Pathogenic Avian Influenza (HPAI) H5N1 virus has dramatically spread throughout Southeast Asia since its first detection in 1997 [1]. Over 560 human cases were reported so far to the World Health Organization (WHO), around 60% of them being fatal [2]. Asia has been the most affected continent, accounting for 73% of the total number of confirmed cases, and 84% of the fatalities worldwide. In Cambodia, a total of 28 outbreaks were reported in poultry since the first detection of the HPAI H5N1 virus in the country in 2004 [3] and 18 confirmed human cases were reported to the WHO, including 16 fatalities [2].

Buddhism is the major religion in several Southeast Asian countries, especially in Laos, Myanmar, Thailand and Cambodia, where 67%, 89%, 95% and 97% of the national population consider themselves as Buddhists respectively, amounting to almost 130 million people [4]. In China, although a lower percentage of the population is officially declared as Buddhist (11–16%), this proportion still represents 200 million people and in reality almost 50% of the Vietnamese are probably practicing this religion [5]. Buddhist life is governed by a number of unassailable rituals. The practice of life releasing is one of the most popular in Southeast Asia. Buddhism considers that all animals are sentient beings, and have, as such, the potential to attain Buddhahood, *i.e.* enlightenment. Besides, according to the Buddhist principle of perpetual reincarnations, any living being has also been at one time one's relative. Thus, among all positive karma (*i.e.* good actions), that of releasing life is considered to be the highest. Many people, in Southeast Asia, are Buddhist followers who purchase those Merit Release Birds (MRBs), mostly Passerine species, in pagodas, raise them in cupped palms to their lips, kiss them, and then release them.

The implications of such ritual in the transmission of zoonotic diseases have not been widely studied before [6], [7]. However, close physical contact between wild birds and humans could be a great risk factor for the transmission of infectious diseases such as avian influenza.

Although wild waterfowl is thought to be a natural reservoir of all influenza subtypes [8], little is known about terrestrial wild birds. Passerine species are known to be susceptible to HPAI H5N1 infection [9]–[16], even though their susceptibility can vary depending on the bird species or the viral strain. Natural infections were also described [12], [17]–[21]. Nonetheless, the exact role of those birds in the natural cycle of H5N1 virus was never determined.

In order to better understand this role, we conducted a number of experiments. We investigated the susceptibility of sparrows to HPAI H5N1 infection, their ability to transmit the virus to chickens, as well as their ability to get contaminated through contact with infected ducks. In parallel, we studied the survival of the virus on infected sparrows' feathers. This study led us to a better understanding of the potential role of the Merit Release Birds in the H5N1 virus natural contamination cycle.

## Methods

### Biosafety and Ethics Statement

All experiments using HPAI H5N1 virus and all animal experiments were carried out in the BioSafety level 3 Laboratory (BSL-3) of the Institut Pasteur in Cambodia, complying with the Animal Committee regulations of Institut Pasteur in Paris, France, in accordance with the EC 86/609/CEE directive, and approved by the Animal Ethics Committee of Institut Pasteur in Cambodia (permit number: AEC/IPC/001/2008).

### Animals

Eurasian Tree Sparrows (*Passer montanus*) were purchased at surrounding Buddhist pagodas in Phnom Penh (Cambodia). They were maintained for a minimum of 5 days for acclimation before inclusion into the experiments. Specific Pathogen Free (SPF) chickens and ducks, of local Cambodian breeds, 4-to-6-weeks old, were kindly provided by the NaVRI. Before inoculation, oro-pharyngeal and cloacal swabs, feathers, as well as blood samples were collected from the birds (all chickens and ducks, and in a subset of randomly selected sparrows of each batch purchased) in order to exclude preexisting exposure to H5N1 virus. All swabs and feathers were stored in viral transport medium (VTM) at −80°C until testing. Blood samples were centrifuged at 3000 rpm for 15 minutes and tested by serology. During the experiments, all birds were housed in self-contained isolation units (Cap Engineering B.V. HM1500 Isolator) ventilated under negative pressure with High-Efficiency Particulate Air (HEPA)-filtered air, with 12 daily hours of artificial lighting. Commercial food and water were provided ad libitum.

### Virus

The HPAI H5N1 virus used in this study was the strain A/Chicken/Cambodia/LC1AL/2007 (GenBank accession numbers HQ200574-200581). The virus stock was obtained after 3 passages in SPF 9-to-11-days old embryonated hen eggs (kindly provided by the National Veterinary Research Institute of Cambodia (NaVRI), Ministry of Agriculture, Forestries and Fisheries (MAFF)) for 48 hours at 37°C. The amnio-allantoic fluid was then harvested and stored at −80°C until further use. Virus titer was determined by calculating the 50% egg infectious dose (EID50) per mL of virus stock. Titration endpoints were calculated using the method of Reed and Muench [22]. To determine the mean lethal dose (LD50) of the virus in sparrows, groups of 5 birds were inoculated via nasal, oral, ocular, and cloacal routes, with serial 10-fold dilution of virus. After the inoculation, sparrows were monitored daily for clinical signs and death for 14 days. The LD50 was calculated by the method of Reed and Muench [22]. The number of RNA copies per mL of virus solution was also determined using the qRT-PCR method described below.

### Experimental design and settings

Seven types of experiments were conducted so as to investigate 3 transmission routes: sparrows' ability to transmit H5N1 infection to each other (A), sparrows' ability to transmit H5N1 infection to chickens (B), and sparrows' ability to be contaminated through contact with infected ducks (C). See Table 1 for details. Inoculations were made on day 0 (D0), and birds exposed to inoculated animals were introduced into the isolators on D1. Birds exposed to H5N1 virus contaminated water were introduced on D0 (Table 1, B.4). Inoculations on birds were made through nasal, ocular, oral and cloacal routes with total volumes of inocula varying from 50 µL (sparrows) to 500 µL (poultry). Different containment conditions were tested for sparrows: they were either left to freely fly around inside the isolator, or contained into a barred cage within the isolator. The standard inoculum dose was defined as 10^6^ EID50 (equivalent to 10^4.63^ LD50 and 10^9.65^ RNA copies) per bird. Two experiments were carried out with a modified inoculum dose of 10^5.23^ EID50, which was determined as representing the total amount of virus shed by 24 infected sparrows during one hour through their feces (Table 1, B.3-B.4). All experiments were conducted twice. For each experiment, a control group was created with an equal number of birds, and the same experimental design was applied using a sham-inoculum.

**Table 1:** Seven different experimental settings.

Principles	#	Animals (or materials) inoculated on Day0	Inoculum dose (EID50)	Animals introduced on Day1	Containment conditions & remarks
Sparrows' ability to transmit H5N1 virus to each other	A.1	Sparrows (n = 8)	10^6^ /sparrow	Sparrows (n = 7)	All sparrows were freed in the isolator
	A.2	Sparrows (n = 10)	10^6^ /sparrow	Sparrows (n = 11)	All sparrows were confined in the same cage within the isolator
Sparrows' ability to transmit H5N1 virus to chickens	B.1	Sparrows (n = 12)	10^6^ /sparrow	Chickens (n = 20)	All birds were freed in the isolator
	B.2.	Sparrows (n = 5)	10^6^ /sparrow	Chickens (n = 6)	The sparrows were caged, whereas the chickens were freed in the isolator
	B.3	Chickens (n = 5)	10^5.23^ /chicken	none	All chickens were freed in the isolator
	B.4	Water	10^5.23^ in 1L of water	none	Chickens (n = 5) were introduced into the isolator containing the contaminated water on D0
Sparrows' ability to be contaminated through contact with infected ducks	C	Ducks (n = 40)	10^6^ /duck	Sparrows (n = 6)	All birds were freed in the whole isolator

After inoculation, oral and cloacal swabs, as well as feathers, were collected daily, and stored in VTM at −80°C until further analysis. All samples collected before and after inoculation/exposition were tested following the protocols described below.

All birds were observed for expression of clinical signs of illness on a daily basis. The main clinical signs expected included: cloudy and/or watery eyes, head and/or face oedema, cyanosis, weakness, anorexia, depression, ruffled feathers, neurological signs (tremors, seizures, incoordination, paralysis, torticollis, etc.), respiratory difficulties, diarrhea, and death. The birds that were still alive 15 days after infection or exposure to contaminated animals or water were killed humanly.

Post-mortem examination and collection of samples were conducted on all animals. The samples collected post-mortem included: blood, oral and cloacal swabs, feathers, and organs such as trachea, muscle, heart, liver, spleen, air sacs, intestines, kidneys, lungs, and brain. All oral and cloacal swabs, and feathers, were stored in VTM at −80°C. All organs were stored in Phosphate Buffer Saline (PBS) at −80°C on one hand, in 10% neutral buffered formalin at room temperature on the other hand.

### RNA extraction, Real-time PCR testing (H5)

Supernatants were collected from swabs stored in VTM and directly processed for RNA extraction. Solid samples, such as feathers and organs, stored in VTM or PBS, went through a homogenization step before RNA extraction, using the MagNa Lyser Instrument (Roche Diagnostics, Mannheim, Germany). RNA was extracted using the MagNa Pure LC Total Nucleic Acid Isolation Kit (Roche) and the MagNa Pure LC Instrument (Roche). The extracted RNAs were tested with quantitative real-time Retro-Transcription and Polymerase Chain Reaction (qRT-PCR) targeting the H5 hemagglutinin gene (H5HA), using the primers H5HA-205-227v2-For (5′ – CGA TCT AGA YGG GGT GAA RCC TC – 3′) and H5HA-326-302v2-Rev (5′ – CCT TCT CCA CTA TGT ANG ACC ATT C – 3′), and the probes H5-Probe-239-RVa (5′ – (Fam) – AGC CAY CCA GCT ACR CTA CA – (MGB) – 3′) and H5-Probe-239-RVb (5′ – (Fam) – AGC CAT CCC GCA ACA CTA CA – (MGB) – 3′ [23], on a Bio-Rad iQ5 Multicolor Real-Time PCR Detection (Bio-Rad). H5 quantified synthetic RNA was used as internal control and to determine the viral load. Viral loads were expressed in number of viral RNA copies per milliliter of supernatants (swabs), or per gram of sample (organs and feathers).

### Serology testing

Serum samples were treated with receptor-destroying enzyme RDE (II) “SEIKEN” (Denka Seiken Co., LTD, Japan) as instructed by the manufacturer, and then heat-inactivated at 56°C for 30 minutes. Hemagglutination inhibition (HI) tests were then performed using 0.5% chicken red blood cells. HI titers were determined as the reciprocal of the highest serum dilution that inhibited the agglutination of 4 hemagglutining units (HAU) of virus. Sera with HI titers ≥16 units were considered positive for the presence of anti-influenza antibodies [24].

### Histopathology and immunohistochemistry

Immunohistochemical staining of the tissues obtained from MRBs only was carried out for the influenza nucleoprotein using HB65 (European Veterinary Laboratories, Netherlands) as described in previously published reports [25].

### Infectious H5N1 particles on sparrows' feathers

Feathers obtained from all sparrows were first homogenized using the MagNa Lyser Instrument (Roche). Each homogenized sample was then inoculated onto three 9-to-11-days old SPF hen eggs and incubated during 48 to 72 hours at 37°C. An hemagglutination (HA) assay was performed on all eggs after each passage, in order to detect infectious H5N1 particles. Positive HA results were further confirmed by qRT-PCR.

## Results

### Susceptibility of the various bird species to direct inoculation of HPAI H5N1 virus

**Figure 1:**
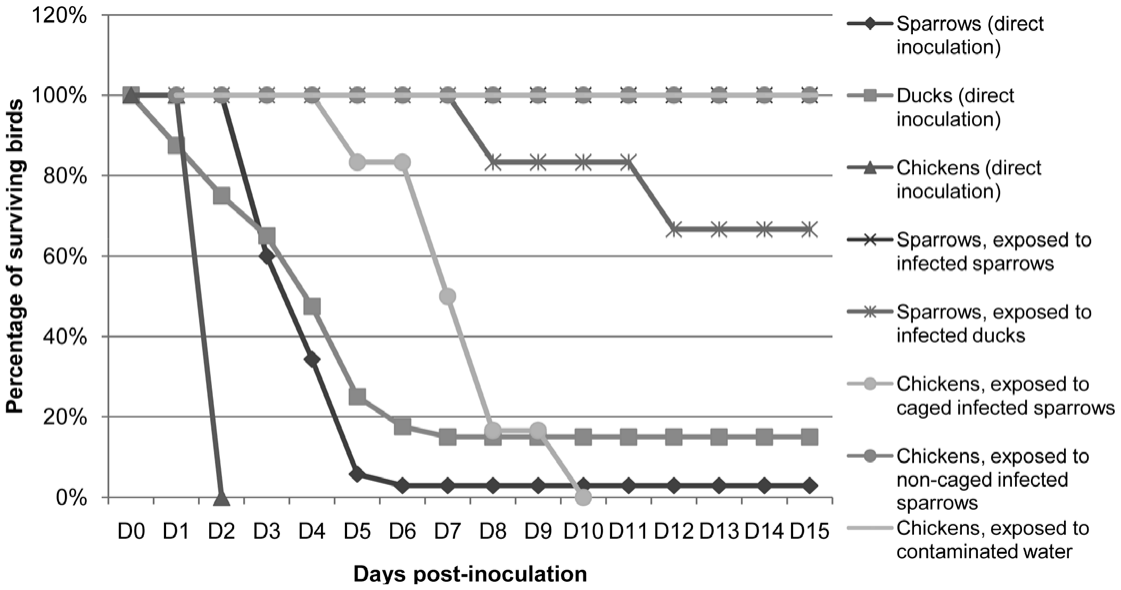
Death kinetics of all birds directly or indirectly exposed to HPAI H5N1 virus.

Sparrows, ducks and chickens were highly susceptible to the HPAI H5N1 strain used when directly inoculated, with overall fatality rates of 97%, 85% and 100% respectively. No symptoms were observed in chickens, which all died on D2 post-inoculation (Figure 1). As for the ducks that died after direct inoculation, the mean death time (MDT) was 3.7 days (Table 2), and all of them experienced neurological signs including tremors, severe incoordination, repeated falls, absence of voluntary movements, pedaling movements, circling, phases of severe despondency, as well as cloudy eyes and facial oedema prior to death. Sparrows infected by direct inoculation, in contrast, exhibited mild clinical signs such as depression, huddling, anorexia, ruffled feathers, and no neurological signs. Except for the few birds which survived the inoculation, death occurred within 4 days on average.

**Table 2:** Mortality rates, mean viral loads.

#‡	Animals infected experimentally						Animals exposed					
	Sp*	FR*	MDT*	MVL* † (no. RNA copies)			Sp*	FR*	MDT*	MVL* † (no. RNA copies)		
				Swab	Feather	Organ				Swab	Feather	Organ
**A.1**	SPR	100	4.1	2.74×10^8^	1.13×10^10^	6.01×10^10^	SPR	0	NA	0	0	0
**A.2**	SPR	100	3.7	2.89×10^6^	1.56×10^6^	3.37×10^9^	SPR	0	NA	0	0	0
**B.1**	SPR	100	4	6.42×10^5^	4.34×10^6^	1.88×10^9^	CK	0	NA	0	0	0
**B.2**	SPR	80	4	1.24×10^7^	8.12×10^2^	6.37×10^9^	CK	100	6.5	1.87×10^7^	1.10×10^8^	1.44×10^10^
**B.3**	CK	100	2	1.35×10^7^	3.16×10^9^	3.30×10^9^	NA	NA	NA	NA	NA	NA
**B.4**	NA	NA	NA	NA	NA	NA	CK	0	NA	0	0	0
**C**	DK	85	3.7	8.15×10^6^	2.35×10^10^	1.74×10^10^	SPR	50	9	1.93×10^6^	2.59×10^8^	5.44×10^9^

*Sp  =  Species (CK  =  Chicken, DK  =  Duck, SPR  =  Sparrow); FR  =  Fatality Rate (%); MDT  =  Mean Death Time (days); MVL  =  Mean Viral Load (no. RNA copies) per mL of swab's supernatant (Swabs), per gram of feathers (Feather), per gram of organ (Organ); NA  =  Not Applicable.

† MVL reported here include data recorded from lethally infected birds only.

‡ All values appearing in the # column correspond to the 7 different experimental settings described in Table 1: A  =  Sparrows' ability to transmit H5N1 virus to each other when freed in the isolator (A.1) or caged (A.2.); B.1 & B.2  =  Sparrows' ability to transmit H5N1 virus to chickens when freed in the isolator (B.1) or caged (B.2); B.3 & B.4  =  Chickens' susceptibility to H5N1 when inoculated with 10^5.23^ EID50, dose determined as representing the total amount of virus shed by 24 infected sparrows during one hour through their feces, through direct inoculation (B.3.) or through exposure to contaminated water (B.4); C  =  Sparrows' ability to be contaminated through contact with infected ducks.

Moderate nephromegaly, splenomegaly, liver discolouration, pancreatomegaly, cholecystomegaly, hyperdilatation of brain blood vessels, hemorrhagic lungs, and pericarditis were among the occasional abnormalities observed during the post-mortem examinations of all bird species used in this study.

Immunohistochemical staining of the tissues confirmed the presence of H5N1 antigen in several Passerine's tissues, namely skeletal and cardiac muscles, trachea, liver and lungs (Figure S1).

**Figure 2:**
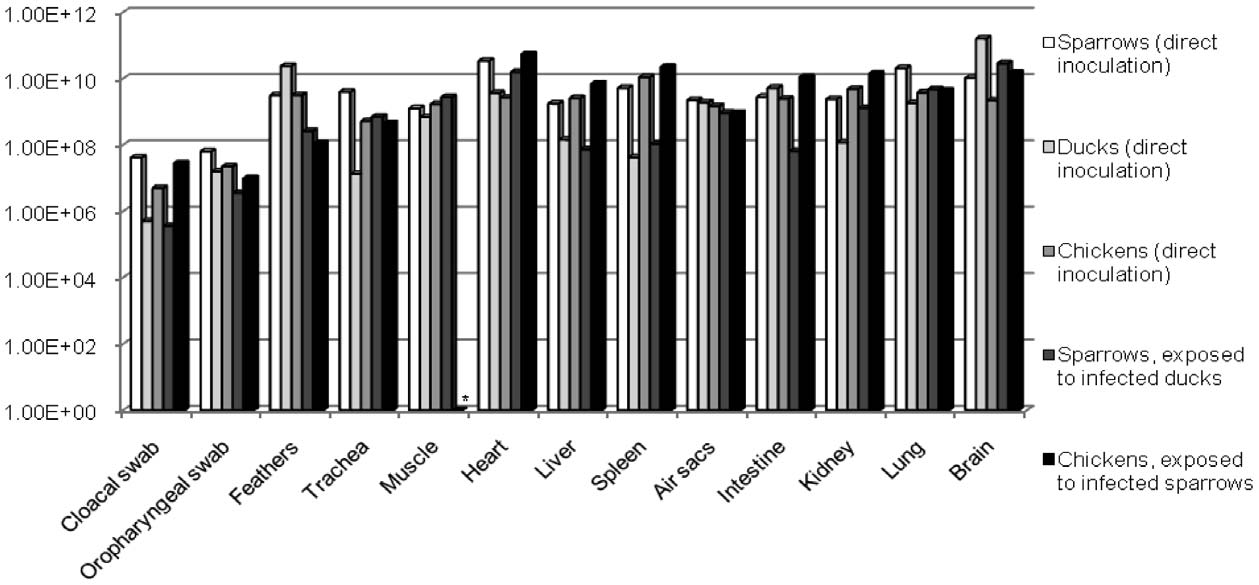
Mean viral loads in samples collected post-mortem. *NA  =  not available. Data presented include only birds that died following H5N1 infection.

High viral loads were detected in all samples collected post-mortem from birds lethally infected through direct inoculation (Figure 2). Swabs' specimens showed mean viral loads of 10^7.72^, 10^6.91^, and 10^7.13^ viral RNA copies per mL of VTM for inoculated sparrows, ducks and chickens respectively. Our observations also allowed us to determine the total amount of virus shed by 24 infected sparrows during one hour through their feces, at 10^5.23^ EID50. This was the concentration used in experiments B.3 and B.4. Mean viral loads found in inoculated sparrows', ducks' and chickens' organs were respectively of 10^9.93^, 10^10.24^, and 10^9.52^ RNA copies per gram on average. As for feathers, respective mean values of 10^9.50^, 10^10.37^, and 10^9.50^ viral RNA copies per gram were determined for sparrows, chickens and ducks.

All surviving birds seroconverted (Table 3).

**Table 3:** Seroconversion rates in surviving birds.

#†	Animals inoculated		Animals exposed	
	Sp*	SR*	Sp*	SR*
**A.1**	SPR	NA	SPR	14
**A.2**	SPR	NA	SPR	36
**B.1**	SPR	NA	CK	0
**B.2**	SPR	100	CK	NA
**B.3**	CK	NA	NA	NA
**B.4**	NA	NA	CK	20
**C**	DK	100	SPR	50

*Sp  =  Species (CK  =  Chicken, DK  =  Duck, SPR  =  Sparrow); SR  =  Seroconversion Rate in surviving birds (%); NA  =  Not Applicable.

† All values appearing in the # column correspond to the 7 different experiments described in Table 1: A  =  Sparrows' ability to transmit H5N1 infection to each other when freed in the isolator (A.1) or caged (A.2.); B.1 & B.2  =  Sparrows' ability to transmit H5N1 infection to chickens when freed in the isolator (B.1) or caged (B.2); B.3 & B.4  =  Chickens' susceptibility to H5N1 when inoculated with 10^5.23^ EID50, dose determined as representing the total amount of virus shed by 24 infected sparrows during one hour through their feces, through direct inoculation (B.3.) or through exposure to contaminated water (B.4); C  =  Sparrows' ability to be contaminated through contact with infected ducks.

### Sparrows' ability to transmit HPAI H5N1 virus to each other (Table 1, A)

**Figure 3:**
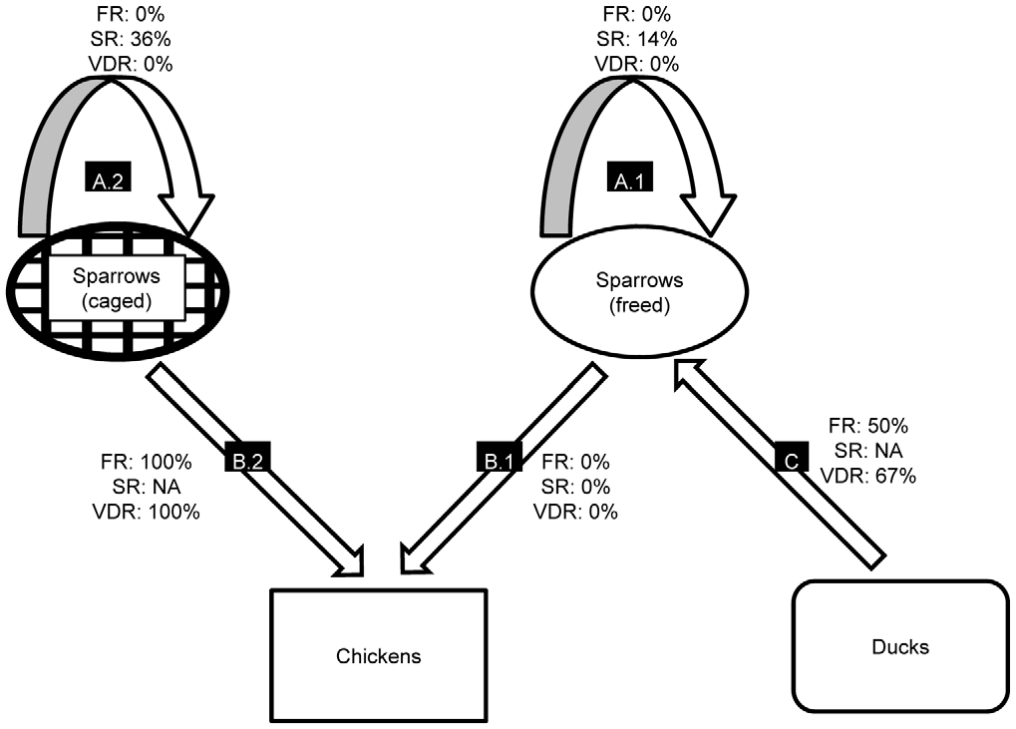
Paths of transmission of H5N1 virus between birds. FR  =  Fatality Rate; SR  =  Seroconversion Rate; VDR  =  Virus Detection Rate; NA  =  not available. Arrows and their respective black-filled rectangles correspond to the different experimental settings described in Table 1: A  =  Sparrows' ability to transmit H5N1 virus to each other when freed in the isolator (A.1.) or caged (A.2.); B.1 & B.2  =  Sparrows' ability to transmit H5N1 virus to chickens when freed in the isolator (B.1) or caged (B.2); C  =  Sparrows' ability to be contaminated through contact with infected ducks.

Regardless of the conditions of containment of the birds, exposure of naïve sparrows to inoculated ones did not lead to any clinical manifestation nor death, despite a 100% mortality rate observed in inoculated MRBs in the A.1 and A.2 experiments (Table 2, A; Figure 3). No virus was detected in any of the samples collected post-mortem from the exposed sparrows. However, seroconversion was observed for 28% of them.

### Sparrows' ability to transmit HPAI H5N1 virus to chickens (Table 1, B)

When left to freely fly within the whole isolator, the sparrows did not transmit H5N1 infection to the chickens (Table 2, B.1). No mortality, no symptoms, no viral RNA, and no seroconversion were observed in any of the exposed chickens. However, transmission did occur when chickens were confined in the isolator with caged sparrows. The clinical symptoms observed included huddling, weight loss, anorexia, and depression. Death occurred in all chickens exposed to caged sparrows within a MDT of 6.5 days post-exposure (Figure 1, Table 2, B.2). Viral loads determined in samples collected post-mortem from those chickens were high, with an average of 10^7.27^ RNA copies per mL for swabs' specimens, 10^8.04^ RNA copies per gram for feathers, and 10^10.16^ RNA copies per gram for organs (Figure 2). Exposure of chickens to water contaminated with sparrows' feces did not lead to any clinical infection (Table 2, B.4). All chickens exposed to contaminated drinking water survived, and no virus was detected in the organs collected during the necropsies. However, anti-H5 antibodies were detected in one chicken's serum, suggesting a subclinical infection (Table 3).

### Sparrows' ability to be contaminated through contact with HPAI H5N1 infected ducks (Table 1, C)

H5N1 viral RNA was detected in 67% of the sparrows exposed to inoculated ducks, with a case fatality rate of 50%. Deadly contaminated sparrows showed high viral loads in all organs (Figure 2), while in the surviving ones, the viral loads were much lower and detected only in some of the organs tested: approximately 10^4.55^ RNA copies per mL for swabs' specimens, 10^5.09^ RNA copies per gram for feathers, and 10^4.72^ RNA copies per gram for organs. Precisely, quantitative RT-PCR tested negative in trachea, brain and liver samples, and tested positive in all other organs.

### H5N1 particles detected on infected sparrows' feathers

Feathers of 94 sparrows were tested. A total of 50 birds tested positive for the presence of viral RNA on their feathers, and 24 were found to carry infectious particles on their feathers.

## Discussion

Here, Eurasian Tree Sparrows were shown to be highly susceptible to the HPAI H5N1 strain used. However, the transmission of the virus between sparrows themselves was quite low, be it released in a wide space (14% of seroconversion) or contained in a narrow cage (36% of seroconversion), and led only to asymptomatic infections with no detectable virus infection or shedding (Table 3, Figure 3), suggesting that introduction by sellers of infected MRBs into the cages together with naïve birds might not lead to efficient infection dissemination. Although the viral loads (∼10^8.44^ viral copies per mL) detected in swabs specimens collected from deadly infected birds correspond to the viral load that would have been measured in 10^3.42^ LD50 of inoculum, this does not mean that all the viruses detected by qRT-PCR were infectious. In addition, the infection of animals is probably less efficient after natural exposure then through experimental inoculation directly in the respiratory and digestive tracts.

Similarly, when exposed to infected sparrows freed in the isolator, no chickens were infected despite their high susceptibility to the strain used (Table 1, B.1). There was no transmission of virus from the sparrows to the chickens, even though the formers did shed virus through cloacal and oral routes and high viral loads were detected on their feathers (Figure 2). The birds were all freely wandering around in the isolators, sharing the same food and water trays. These confinement conditions were meant to simulate, as faithfully as possible - considering the biosafety constraints - real field conditions. In the isolators, sparrows were provided with a high-located bar so that they could stay away from the chickens when needed, as they would in natural conditions.

Nonetheless, on the other hand, when caged and unable to escape from the chickens, transmission occurred. The caging, instead of protecting and separating the sparrows from the chickens, made them more vulnerable. They were placed in a stressful situation, in which chickens perpetually harassed them through the bars, and were able to easily peck them. This behavior led to the chickens' infection.

In order to better understand the absence of H5N1 virus transmission from sparrows to chickens when close contacts are not forced, we tried to evaluate the level of exposure of the chickens to the avian influenza virus during the experiment. We estimated that 24 infected sparrows shed through feces the amount of 10^5.23^ EID50 virus per hour. We considered these conditions as extreme since the probability of having 24 infected sparrows spending one full hour dropping their feces either directly into a chicken's external orifices, or into the same liter of water, was highly unlikely. Our findings demonstrated that even at this dose, the seeded drinking water was not able to cause clinical disease in chickens, whereas the same dose was proved to be lethal when directly inoculated to chickens through nasal, ocular, oral and cloacal routes (Table 2, B.3-B.4). Thus, these results suggest that MRBs might not be able to shed a sufficient quantity of H5N1 virus in the environment to infect poultry in field conditions, unless carcasses of sparrows infected by H5N1 virus are pecked at, *i.e.* scavenged by chickens. Indeed, while viral particles shed by infected sparrows in the environment through their feces might be at too low a concentration to efficiently infect the poultry [26], chickens' common propensity for pica could result in the scavenging of highly infectious carcasses, and thus allow the virus to spread from one host to another and from place to place. Infected sparrows carcasses might also be eaten by carnivores or by various scavenger birds species which may also contribute to the virus dissemination.

Efficient viral transmission also occurred from ducks to sparrows. Ducks are often incriminated in the silent spread of influenza A viruses, because of their frequent ability to undergo asymptomatic infections [27]–[31]. Thus, our data suggest that in areas where H5N1 virus is circulating in domestic birds, sparrows might get infected through contact with infected poultry, which could probably explain most of the natural contaminations of sparrows documented in the literature [12], [17]–[21]. It is noteworthy that it took around 9 days for these sparrows to die of H5N1 infection after close contact with infected ducks (Table 2, C). The hypothesis of having infectious sparrows flying from farm to farm or being carried from one place to another for religious purposes, while having repeated contacts with humans therefore cannot be ruled out, especially as our study shows that infected sparrows display usually relatively few clinical symptoms.

Several infected sparrows were carrying infectious particles on their feathers. The presence of H5N1 virus antigens in feather follicles, associated with histological lesions, was first assessed and described by Perkins and Swayne in 2001 [32]. Since then, some studies have explored the role of feathers in HPAI virus cycles [33]–[39]. These studies showed the presence and persistence of infectious particles in feathers as long as 5 days after the death of the animal [33]. Evidence of the presence of viable particles on feathers was also provided from asymptomatic birds [37]. Thus, the potential role of infected feathers in the transmission cycle of H5N1 virus should not be neglected in the context of popular Buddhist rituals, for which birds are to be manipulated and even kissed. In addition, one H5N1 virus isolated from a tree sparrow in Indonesia was recently found to be genetically closely related to human viruses previously detected in the same island, suggesting that humans and MRBs are susceptible to similar strains of the virus [20]. Thus, even though sparrow-to-human transmission of H5N1 virus was never documented, such transmission is in theory possible and MRBs might indeed represent a potential risk for human and animal contamination.

## Supporting Information

Figure S1 **Immunohistochemical analysis for H5N1 virus nucleoprotein detection in merit release birds' organs (experimentally infected).** Immunohistochemical staining of the tissues was carried out for the influenza nucleoprotein detection in sparrows' tissues. A) Lung section: arrows are pointing at few infected cells (red-purple). B) Liver section: numerous influenza-infected cells appear in red.
(PPT)
