# Supplementary material for: Eurasian Tree Sparrows, Risk for H5N1 Virus Spread and Human Contamination through Buddhist Ritual: An Experimental Approach
Source: PLoS One. 2011 Dec 2;6(12):e28609. doi: 10.1371/journal.pone.0028609 (PMC3229601; doi:10.1371/journal.pone.0028609)
Supplement: Figure S1 — Immunohistochemical analysis for H5N1 virus nucleoprotein detection in merit release birds' organs (experimentally infected). Immunohistochemical staining of the tissues was carried out for the influenza nucleoprotein detection in sparrows' tissues. A) Lung section: arrows are pointing at few infected cells (red-purple). B) Liver section: numerous influenza-infected cells appear in red. (PPT) [file pone.0028609.s001.ppt]

## Slide 1
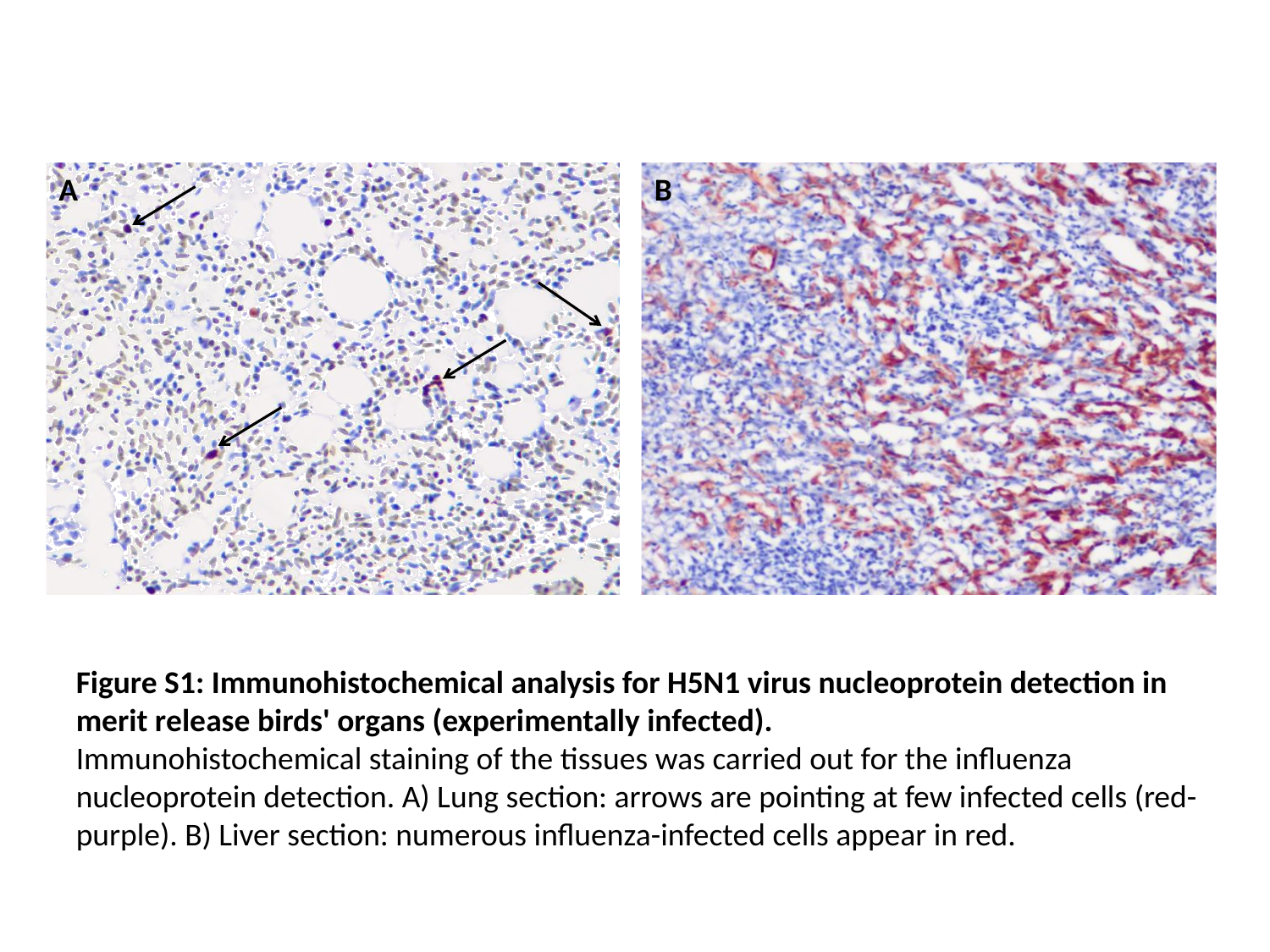

A
B
Figure S1: Immunohistochemical analysis for H5N1 virus nucleoprotein detection in merit release birds' organs (experimentally infected).
Immunohistochemical staining of the tissues was carried out for the influenza nucleoprotein detection. A) Lung section: arrows are pointing at few infected cells (red-purple). B) Liver section: numerous influenza-infected cells appear in red.
